# Abstracts

**DOI:** 10.1155/S1463924684000341

**Published:** 1984


### Abstracts

### Page 175

### A multidimensional approach to analytical science—from invention and prototypes to business on an international scale (1939–1976)

R. Stanley

Invention so often occurs at the boundaries between two or more separate disciplines. Out of this kind of background the concept of the continuous-flow analysis was born. By chance, the Technicon Company in America were fortunate to be introduced to Leonard T. Skeggs, Jr and his analytical system. They adopted the system and made it commercially available under the name of the AutoAnalyzer. It was protected by a strong patent. Through sound business skill and expertise in the technical fields, the system won world-wide recognition, and as a result realized staggering financial success. Particular factors contributing to that success are reviewed in this article. These factors are considered in a conclusion with respect to the instrument business in Britain.

### Une approche multidimensionnelle à la science analytique—de l'invention et des prototypes aux affaires à niveau international (1939–1976)

R. Stanley

Les inventions sont souvent à cheval entre deux disciplines plus ou moins séparées. Sur cet arrière-plan la conception de l'analyse à flux continu a été inventée. Tout par hasard la compagnie Technicon aux Etats-Unis a eu la chance d'être introduite à Leornard T. Skeggs, jr. et son système analytique. Cette compagnie a adopté le système et l'a commercialisé sous le nom de AutoAnalyzer. Il était protégé par des brevets puissants. Grâce à une grande expertise d'affaires et dans le domaine technique, le système a gagné une bonne reconnaissance mondiale et, en conséquence, a réalisé un grand succés financier. Les facteurs particuliers contribuants à ce succès sont revus dans cet article. Ces facteurs sont considérés en conclusion en comparaison avec le commerce d'instruments en Grande Bretagne.

### Ein multidimensionaler Ansatz zur Analytik—von der Erfindung und dem Prototyp zum Geschäft in internationalem Ausmass (1939–1976)

R. Stanley

Erfindungen erfolgen oft an der Grenze zwischen zwei oder mehreren Disziplinen. Auf diesem Hintergrund entstand das Konzept der kontinuierlichen Durchflussanalyse. Durch einen glücklichen Zufall wurde die Firma Technicon in den USA auf Leonard T. Skeggs Jr. und sein analytisches System aufmerksam. Sie übernahmen das System und brachten es unter der Bezeichnung AutoAnalyser auf den Markt. Es war durch ein starkes Patent geschützt. Dank einer geschickten Geschäftstätigkeit und technischer Kompentz gewann das System weltweite Anerkennung und resultierte in einem gewaltigen finanziellen Erfolg. Spezielle Faktoren, die zu diesem Erfolg beitrugen, werden in dieser Uebersicht besprochen. Diese Faktoren werden in der Schlussfolgerung einer Betrachtung in Bezug auf das Instrumentengeschäft in Grossbritannien unterzogen.

---

### Page 186

### An automated microprocessor-based spectrophotometric flow-injection analyser

M. Koupparis and P. Anagnostopoulou

An inexpensive, fully automated spectrophotometric flow-injection analyser using common laboratory instruments is described. A microcomputer is used to control such function as sampling, injection, peak measurement and calculation. The base-line is continuously measured before each set of injections, ensuring a high, long-term system stability. The performance of the system was evaluated with a simple dilution of a dye (2,6-dichlorophenol-indophenol, DCPI), an iron (III) determination with thiocyanate ions and an ascorbic acid determination with DCPI. In all cases high precision (better than 0·8% RSD), excellent linearity, high long-term stability and high measurement throughputs (80–200/h) were obtained.

### Un analyseur spectrophotométrique à injection de flux automatisé et basé sur microprocesseur.

M. Koupparis and P. Anagnostopoulou

Un analyseur spectrophotométrique à injection de flux complètement automatisé et bon marché utilisant des instruments de laboratoire communs est décrit. Un miniordinateur est utilisé pour contrôler les fonctions telles que l'échantillonnage, l'injection, la mesure des pics et le calcul. La ligne de base est mesurée continuellement avant chaque série d'injections assurant une haute stabilité du système à long terme. La performance du système a été évaluée avec une simple dilution d'un colorant (2,6-dichlorophenol-indophenol, DCPI), une détermination de fer (III) avec des ions thiocyanate et une analyse de l'acide ascorbique avec DCPI. Dans tous les cas, une haute précision (meilleure que 0·8% d'écart type), une excellente linéarité, une bonne stabilité à long terme et un haut passage de mesures (80–200/h) ont été obtenus.

### Ein automatisiertes spektrophotometrisches FIA-System mit Mikroprozessor

M. Koupparis and P. Anagnostopoulou

Ein kostengünstiges, vollautomatisiertes spcktrophotometrisches FIA-Analysensystem wird beschrieben, das nur gewöhnliche Laborinstrumente verwendet. Ein Mikrocomputer steuert Funktionen wie Probenziehen, Einspritzen, Kurvenvermessung und Signalverarbeitung. Die Basislinie wird vor jedem Satz von Einspritzungen kontinuierlich vermessen und sichert damit eine hohe Langzeitstabilität des Systems. Die Leistungsfähigkeit des Systems wurde mit einer einfachen Verdünnung eines Farbstoffes (2,6-Dichlorphenolindophenol, DCPI), einer Eisen (III)-Bestimmung mit Thiocyanat-Ionen und einer Ascorbinsäure-Bestimmung mit DCPI evaluiert. In allen Fällen wurde eine hohe Präzision (besser als 0·8% RSA), ausgezeichnete Linearität, hohe Langzeitstabilität und ein hoher Durchsatz (80–200 Messungen pro Stunde) erhalten.

---

### Page 192

### A flexible computer-to-computer protocol for DISNET: a Distributed Instrument System NETwork

P. J. Gemperline and R. Megargle

This paper describes a software system which implements a network protocol for the Distributed Instrumentation System

NETwork, DISNET. The network software has been integrated into the host computers to provide application programs with a powerful set of operating system calls. These calls can be used to dynamically create and control up to seven separate communication channels per host. The combined hardware and software systems bring the unique advantage of a truly distributed system to the laboratory environment.

### Un protocole flexible d'ordinateur à ordinateur pour DISNET: Un réseau d'instruments distribués

P. J. Gemperline and R. Megargle

Cette publication présente un système de logiciel qui réalise un protocole pour le réseau d'instruments distribués, DISNET. Le logiciel de réseau a été intégré en un ordinateur pour procurer des programmes d'applications avec possibilités puissantes d'appel du système opérationnel. Ces appels peuvent être utilisés pour créer et contrôler jusqu'à 7 canaux de communication séparés par ordinateur. Le système combiné de matériel et logiciel apporte l'avantage unique d'un vrai système distribué pour l'environnement du laboratoire.

### Ein flexibles Computer/Computer Protokoll für DISNET: Neztwerksystem für den Anschluss verteilter Instrumente

P. J. Gemperline and R. Megargle

Diese Arbeit beschreibt ein Softwaresystem, das ein Netzwerkprotokoll für das Distributed Instrumentation System NETwork, DISNET, darstellt. Die Netzwerksoftware wurde in die übergeordneten Rechner integriert, um damit Applikationsprogramme mit einem leistungsfähigen Satz von Befehlen an das Betriebssystem zu versehen. Diese Befehle können dazu verwendet werden, um damit dynamisch bis zu sieben verschiedenen Kommunikationskanäle pro übergeordneten Rechner zu schaffen und zu kontrollieren. Die kombinierten Hardware/Softwaresysteme bringen die einzigartigen Vorteile eines echten verteilten Systems in den Bereich des Labors.

---

### Page 197

### The implementation of a GC/MS data system using DISNET: a Distributed Instrument System NETwork

P. J. Gemperline and R. Megargle

A resource sharing protocol has been designed for the Distributed Instrument System NETwork (DISNET) to support a distributed processing environment for real-time instrument control, data acquisition, and computation. A prototype disk resource provider using the resource sharing protocol has been developed. Design features of the disk resource include block structured input/output for random access files, resource provider/consumer synchronization, and protection mechanisms from third-party consumers. A distributed gas chromatography/mass spectrometry (GC/MS) data system has been implemented using the DISNET resource sharing protocol which provides the usual features found on quadrupole data systems, as well as a unique application of distributed processing in a real-time environment. Examples are given to demonstrate the capabilities of the GC/MS data system.

### La mise en oeuvre d'un système de données pour un GC/MS utilisant DISNET: Réseau pour système d'instruments distribués

P. J. Gemperline and R. Megargle

Un protocole se basant sur les mêmes ressources a éré créé pour le réseau pour systèmes d'instruments distribués (DISNET) afin de soutenir un environnement de traitement de données distribuées pour le contrôle d'instrument en temps réel, l'acquisition de données et l'exploitation. Un disque prototype utilisant le protocole de partage de ressources a été développé. La conception du disque inclut une entrée/sortie structurée en blocs pour fichier à accès libre, synchronisation ressource utilisateur et un mécanisme de protection contre des utilisateurs non-autorisés. Un système de données distribuées pour chromatographie en phase gazeuse/spectrométrie de masse (GC/MS) a été installé utilisant le protocole de partage de ressource DISNET qui procure les propriétés habituelles qu'on trouve dans des systèmes de données quadrupoles ainsi qu'une application unique de traitement distribué en un environnement temps réel. Des exemples sont donnés pour démontrer les possibilités du système de données GC/MS.

### Einrichtung eines GC/MS Datensystems mit DISNET: Ein verteiltes Instrumente-Netzwerksystem

P. J. Gemperline and R. Megargle

Für das verteilte Instrumente-Netzwerksystem (DISNET) wurde ein Protokoll für die gemeinsame Benützung aller Geräte implementiert, um damit ein verteiltes Prozesssystem für Echtzeit-Instrumentesteuerung, Datenerfassung und -verarbeitung zu betreiben. Dazu wurde ein Prototyp eines Plattenspeicher-Zuteilsystems entwikkelt, der dieses Protokoll benutzt. Eigenschaften dieses Zuteilsystems sind blockstrukturierter Ein- und Ausgang für ide Random Access Files, Zuteilsystem/Benützer-Synchronisation und Schutzmechanismen gegen Drittbenützer. Mit diesem DISNET-Protokoll wurde ein verteiltes GC/MS Datensystem implementiert, welches die üblichen Eigenschaften eines Quadrupol-Datensystems aufweist, daneben aber eine neuartige Anwendung der verteilten Verarbeitung in einer Echtzeit-Umgebung darstellt. Es werden Beispiele angeführt, die die Leistungsfähigkeit des GC/MS Datensystems demonstrieren.

---

### Page 202

### A microcomputer system for use with an EPR spectrometer

M. J. Adams and G. J. Ewen

An apple IIE microcomputer system has been interfaced to a Varian E104-A spectrometer. Data logging from the spectrometer is achieved with the aid of a 12-bit analogue to digital converter. Recorded data may be computer manipulated and displayed either on a high-resolution graphics monitor or on the spectrometer's pen recorder.

### Un système à microordinateur pour utilisation avec spectromètre EPR

M. J. Adams and G. J. Ewen

Un miniordinateur Apple IIE a été interfaçé à un spectromètre Varian E104-A. Réception des données du spectromètre est réalisée à l'aide d'un convertisseur analogique digital à 12 bits. Les données enregistrées peuvent être manipulées par l'ordinateur et affichées soit sur le moniteur graphique à haute résolution ou sur l'enregistreur du spectromètre.

### Ein Mikrocomputersystem für die Verwendung mit einem EPR Spektrometer

M. J. Adams and G. J. Ewen

Ein Apple IIE Mikrocomputersystem wurde an ein Varian E104-A Spektrometer angeschlossen. Die Erfassung der Daten des Spektrometers wurde mit Hilfe eines 12-bit analog/digital-Wandlers erreicht. Die gespeicherten Daten können mit dem Computer weiter verarbeitet werden und entweder auf einem hochauflöslichen Graphikbildschirm oder auf dem Schreiber des Spektrometers aufgezeichnet werden.

---

### Page 206

### Turbidimetric analysis on the Hitachi 705 using orosomucoid as a model

E. F. Legg and M. Sullivan

The Hitachi 705 programmable discrete analyser has not yet been recommended for the performance of turbidimetric assay. This paper describes the optimization of a turbidimetric analysis for orosomucoid and its adaptation for use on the Hitachi 705. The proposed assay is cheap, reliable and rapid and could be extended to enable other specific proteins to be analysed using the Hitachi 705.

### Analyse turbidimétrique avec le Hitachi 705 utilisant comme modèle l'orosomucoïde

E. F. Legg and M. Sullivan

L'analyseur discret et programmable Hitachi 705 n'a pas encore été recommandé pour son emploi comme analyseur turbidimétrique. Cette publication décrit l'optimisation d'une analyse turbidimétrique de l'orosomucoïde et son adaptation sur analyseur Hitachi 705. L'analyse proposée est bon marché, sûre et rapide et peut être généralisée pour permettre d'autres analyses de proteïnes spécifiques utilisant le Hitachi 705.

### Turbidimetrische Analyse auf dem Hitachi 705 mit Verwendung von Orosomucoid als Beispiel

E. F. Legg and M. Sullivan

Der Hitachi 705 programmierbare, diskontinuierliche Analysator wurde bis jetzt für die Ausführung von turbidimetrischen Analysen nicht empfohlen. Die Arbeit beschreibt die Optimierung einer turbidimetrischen Analyse für Orosomucoid und deren Anpassung im Gebrauch mit dem Hitachi 705. Die vorgeschlagene Analyse ist billig, zuverlässig und schnell und kann in Verwendung mit dem Hitachi 705 erweitert werden, um andere spezifische Proteine zu analysieren.

---

### Page 210

### A microcomputer-based automated curve tracer for accurate digitization of paper-recorded spectra

J. C. W. G. Bink and H. A. van't Klooster

Using an Apple II microcomputer, a Calcomp 81 plotter and a high resolution optical reflective sensor, a low-cost automated curve-tracer is developed. The software is written in CP/M oriented COMPAS-Pascal and includes programs for accurate digitization, smoothing and replotting of the digitized spectra. The method has been tested on 150 infra-red spectra, which were recorded on paper documents. By measuring a maximum of 2400 points on a full infra-red spectrum, the configuration used yields an accuracy of $1\,cm^{-1}$ in the range $4000–2000\,cm^{-1}$ and $0,5\,cm^{-1}$ in the range $2000–600\,cm^{-1}$, at a digitization rate of 15 to 20 min per spectrum. Replots of the digitized spectra are visually hardly distinguishable from the originals.

### Un traceur de courbes automatique basé sur microordinateur pour la digitalisation exacte de courbes tracées sur papier

J. C. W. G. Bink and H. A. van't Klooster

Utilisant un microordinateur Apple II, une imprimante Calcomp 81 et un senseur par réflection optique à haute résolution, un traceur automatique à faible prix a été développé. Le logiciel est décrit en COMPAS-PASCAL orienté vers CP/M et inclut des programmes pour digitalisation exacte, filtrage et retraçage des spectres digitalisés. La méthode a été testée avec 150 spectres infrarouges, qui étaient enregistrés sur des documents sur papier. En mesurant un maximum de 2400 points pour un spectre infrarouge complet, la configuration procure une exactitude de $1\,cm^{-1}$ dans le domaine $4000–2000\,cm^{-1}$ et de $0·5\,cm^{-1}$ dans le domaine $2000–600\,cm^{-1}$, ceci avec une vitesse de digitalisation de 15 à 20 minutes par spectre. A vue d'oeil, les spectres digitalisés et retracés ne se distinguent pratiquement pas des originaux.

### Ein automatisches Kurvenfolgesystem mit Mikrocomputer für die genaue Digitalisierung von analog aufgezeichneten Spektren

J. C. W. G. Bink and H. A. van't Klooster

Unter Verwendung eines Apple II Computers, eines Calcomp 81 Plotters und eines hochauflösenden optischen Reflexionssensors wurde ein kostengünstiges Kurvenfolgegerät entwickelt. Die Software ist in COMPAS-Pascal unter CP/M geschrieben und umfasst Programme für die präzise Digitalisierung, Glättung und Wiederausgabe der digitalisierten Spektren. Die Methode wurde mit Hilfe von 150 Infrarotspektren, welche analog auf Papier aufgezeichnet waren, getestet. Unter Messung von maximal 2400 Punkten pro volles Infrarotspektrum ergab die Konfiguration eine Genauigkeit von $1\,cm^{-1}$ im Bereich $4000–2000\,cm^{-1}$ und $0·5\,cm^{-1}$ im Bereich $2000–600\,cm^{-1}$, bei einer Digitalisierungsgeschwindigkeit von 15–20 Minuten pro Spektrum. Die Aufzeichnungen der digitalisierten Spektren sind visuell kaum unterscheidbar von den Originalen.

---

### Page 214

### The Abbott TDx evaluated for T-uptake

G. A. Harff

A replication experiment and a comparison of methods experiment was performed for T-uptake assay on the Abbott TDx. The procedure of the 'midi' replication experiment, as described in the National Committee for Clinical Laboratory Standards (NCCLS) proposed standard PSEP-3, was followed. For the comparison of methods experiment, the proposed standard PSEP-4 was followed.

The replication experiment was performed with three levels of controls, 28·8% uptake, 37·3% uptake and 51·0% uptake, within-run coefficients of variation were respectively, 2·53%, 2·31% and 3·37%. Total imprecision was found to be 3·31%, 2·52% and 4·45% respectively. Within-run imprecision calculated from patient duplicates divided into three levels, 27·07%, 30·26% and 35·87%, was, respectively, 2·31%, 2·25% and 2·27%.

At $p = 0·05$ a percentage of carry-over was found not to be significant.

Comparison of methods with a T3-uptake RIA method ($x$ axis), comparative method, and the T-uptake on TDx ($y$ axis) resulted in $y = 0·881\ x + 5·08\%$.

### Le TDx d'Abbott évalué pour la fixation de T

G. A. Harff

Une expérience de répétition et de comparaison de méthodes a été réalisée pour la détermination de la fixation T sur le TDx Abbott. Les procédures pour une expérience moyenne de répétition ont été suivies telles que décrites dans le standard proposé PSEP-3 du Comité National pour Standards de Laboratoires Cliniques (NCCLS). Pour l'expérience de comparaison de méthodes le standard proposé PSEP-4 a été suivi.

Expérience de répétition a été faite avec trois niveaux de contrôle, 28·8% fixation, 37·3% fixation et 51·0% fixation résultant en des coéfficients de variation en série de respectivement 2·53%, 2·31%, 3·37%. L'imprécision totale trouvée était de 3·31%, 2·52% et 4·45%. L'imprécision en série calculée de duplicats de malades divisée en trois niveaux, 27·07%, 30·26% et 35·87% était respectivement 2·31%, 2·25% et 2·27%.

A $p=0{\cdot}05$ le pourcentage de contamination trouvé n'était pas significatif. Comparaison de la méthode avec une méthode RIA fixation T3 ($x$) qui est la méthode de comparaison et la fixation T sur le TDx ($y$) résultait en $y=0{\cdot}881\ x+5{\cdot}08\%$.

### Evaluation des Abbott TDx auf die T-Aufnahme

G. A. Harff

Für den T-Aufnahmetest auf dem Abbott TDx wurde ein Replikations experiment und ein Methodenvergleichsexperiment durchgeführt. Das Vorgehen beim Midi-Replikationsexperiment entsprach dem Standard PSEP-3, wie er in den National Committee for Clinical Laboratory Standards (NCCLS) vorgeschlagen wird. Das Vorgehen beim Methodenvergleichs-experiment entsprach dem vorgeschlagenen Standard PSEP-4.

Das Replikationsexperiment wurde mit drei Stufen der Kontrolle durchgeführt: 28·8%, 37·3% und 51·0% Aufnahme. Die Variationskoeffizienten innerhalb einer Serie betrugen 2·53%, 2·31% bzw. 3·37%. Die Gesamtpräzision betrug 3·31%, 2·52% bzw. 4·45%. Die Präzision innerhalb einer Serie, berechnet von auf die drei Stufen 27·07%, 30·26% und 35·87% aufgeteilten Patienten-Duplikaten, betrug 2·31%, 2·25% dzw. 2·27%.

Bei $p=0{\cdot}05$ wurde keine signifikante Verschleppung gefunden. Ein Vergleich der Methoden mit einer T3-Aufnahme RIA-Methode (x-Achse), einer Vergleichsmethode, und der T-Aufnahme auf dem TDx (y-Achse) resultierte in $y=0{\cdot}881\ x+5{\cdot}08\%$.

---

lished in our last issue; we apologize for the delay.

### Vol. 6, No. 3, page 152

### Détermination automatique enzymatique des acides gras libres du plasma par analyse centrifugale

D. P. Knox and D. G. Jones

Une microméthode enzymatique pour la détermination rapide de routine des acides gras libres (FFA) dans le plasma ou le sérum utilisant un analyseur microcentrifugal Multistat III I.L. est décrite. Vu à part les avantages de la vitesse (18 tests/5 min) et sa facilité d'utilisation la méthode de blanc bicromatique dans l'échantillon et de petits volumes de réactifs (240 ml/test) en font une méthode extrêment économique comparée avec les méthodes actuelles en vigueur.

Les conditions de réaction ont été optimalisées afin de donner une réponse linéaire pour les concentrations de FFA dans le plasma entre 0·1 et 2·0 mmol/litre. Les coéfficients de variation en série et entre-séries étaient systématiquement inférieurs à 5% et la corrélation avec la méthode manuelle colorimétrique est bonne. Les valeurs FFA dans le plasma traité avec de l'oxalate/fluorure ou de l'EDTA comme anticoagulant et le sérum étaient similaires, mais la présence de 'héparine provoquait des valeurs trop hautes d'environ 10%. Les acides gras libres dans le plasma ou le sérum étaient stables pour au moins deux semaines à moins −20°C et pour au moins deux jours à 4°C, mais étaient instables àtempérature ambiante.

### Automatisierte enzymatische Bestimmung von plasmafreien Fettsäuren mit Zentrifugalanalyse

D. P. Knox and D. G. Jones

Die Arbeit beschreibt eine enzymatische Mikromethode für die rasche Routinebestimmung von freien Fettsäuren (FFA) in Plasma oder Serum auf einem IL Multistat III Mikrozentrifugalanalysator. Neben den Vorteilen der Geschwindigkeit (18 Teste in 5 Min.) und der Bequemlichkeit ist die Methode wegen der Verwendung der Bichromat-Ausblendung in der Probe und wegen der geringen Reagensvolumina (240 $\mu$l pro Test) im Vergleich mit existierenden manuellen Methoden äusserst kostengünstig.

Die Reaktionsbedingungen wurden auf eine lineare Response auf Plasma FFA-Konzentrationen im Bereich 0·1–2·0 mmol/l optimiert. Die Variationskoeffizienten innerhalb einer Serie und von Serie zu Serie waren stets kleiner als 5%, und die Korrelation mit der manuellen kolorimetrischen Methode war gut. Plasma, mit Oxalat/Fluorid oder EDTA als Antiloagulant versetzt, und Serum gaben ähnliche FFA Werte. Hingegen verursachte die Präsenz von Heparin einen etwa um 10% zu hohen Wert. FFA in Plasma oder Serum ist bei −20°C mindestens während zwei Wochen, bei +4°C mindestens während zweier Tage stabil, aber unstabil bei Zimmertemperatur.

### FORTHCOMING PAPERS

*Articles in the journal in 1985 include:*

David T. Rossi and Harry L. Purdue—An inexpensive fast memory module for rapid acquisition of digital data

W. Bablok and H. Passing—Application of statistical procedures in analytical instrument testing

B. H. Venter and M. L. Siebert—A comprehensive laboratory automation system

P. Henry and D. C. Ephraim—'Instant' cusums from a discrete analyser

T. G. Pellar *et al.*—The clinical biochemistry laboratory computer system as a simple calculator: a program in MUMPS

M. L. Gozzo *et al.*—Use of NEDD as secondary calibrator for conjugated bilirubin on the Du Pont aca

M. S. Sheya and C. Riley—The use of a microprocessor for flexible automation of an experimental procedure

F. Parri and E. de Majo—Evaluation of an automated haemolytic method for the determination of anti-Streptolysin O antibodies

Peter Stockwell—The changing role of computers in the laboratory

M. Legrand and P. Bolla—A fully automatic apparatus for chemical reactions on the laboratory scale

M. Pécs *et al.*—Automatic analyser /computer system for adaptive control of phosphate concentration during fermentation

Jan Hemel—Critical discussion on a method for derivation of reference limits in clinical chemistry from a patient population

*Future topics*

The editor intends publishing a number of articles on robotics in the laboratory and on LIMS (Laboratory Information Management Systems) in late 1985/early 1986. On the latter, he would appreciate suggestions from authors for papers describing evaluations of commercial set-ups or descriptions of user laboratories building their own.

